# Book Reviews

**Published:** 1859-04

**Authors:** 


*Anatomy, Descriptive and Surgical.*—By HENRY GRAY, F. R. S. Lecturer on Anatomy, at St. George's Hospital. The Drawings by H. V. Carter, M. D., &c., imp. 8vo. John W. Parker & Son, London, 1858.

WE have just received a copy of this elegant work, and we take great pleasure in mentioning some points connected with it. Indeed as Anatomy is taught so thoroughly at this time, in the Baltimore

College of Dental Surgery, and as this study is of so much importance to all dental students, we conceive it our duty to assist to the extent of our ability, in making known the best works on the subject. The book before us, is of the very highest excellence in every respect. "It is intended," as the preface informs us, and as we are able to learn from a somewhat careful examination, "to furnish the student and practitioner with an accurate view of the anatomy of the human body, and more especially, the application of this science to practical surgery." As *osteology* forms the basis of all sound anatomical knowledge, we find that much care has been in this work devoted to its treatment. A concise description of the anatomy of the bones, illustrated by numerous accurately lettered engravings, showing the markings, &c. of the bones, has been given. The articulations are carefully studied and illustrated by engravings taken from recent dissections. The muscles and faciæ are described as usual in groups. But one important peculiarity is, that the series of illustrations, to this part, show the lines of incision necessary in the dissection of the muscles in each region. The surgical anatomy of the muscles is also described in connection with fractures. In conclusion, we have only to say, that this work, as far as mechanical exsection goes, does credit, even to the London publishers.

*Contributions to Operative Surgery, and Surgical Pathology*, by J. M. Carnochan, Prof. of Surgery in the N. Y. Medical College, etc. With illustrations drawn from nature. 4to. parts I & II. Lindsay & Blakiston, Phila., 1858.

We would particularly recommend this extremely valuable work, now in course of publication, to such of our readers as desire to be acquainted with the latest achievements of modern surgical skill, and who are anxious to be skillful surgeons. As Prof. Carnochan remarks:—"These contributions . . . . . will be found useful by those who recognize, that it is from the study of particular facts, operative surgery frequently derives its most valuable suggestions. The life of man is too short to allow him, even with the greatest industry, to witness all the varieties of accident or disease; and, as there is no limit to the multiplicity of pathological differences, the most experienced practitioner may find himself in the presence of unknown and unforeseen conditions. Hence, it is incumbent on the surgeon to read much, as well as to see much, and to extend his knowledge, as far as possible, beyond the sphere of his own immediate observation." As will be seen from the "contents," several of the contributions are of great importance to the intelligent dentist: Part I. Case of amputation of the entire lower jaw. Remarks on amputation of lower jaw. Elephantiasis arabum. Part II. Case of exsection of the entire ulna. Remarks on neuralgia of the face, with a case. Exsection of the trunk of the second branch of the fifth pair of nerves beyond the ganglion of Meckel, for severe neuralgia of the face; with three cases. The last contribution will be found in the present number of the Journal.

*Trials of a Public Benefactor*, as illustrated in the Discovery of Etherization. By. Nathan P. Rice, M. D.; 12mo.; pp. 460. New York, 1859.

In calling the attention of our readers to this little book, which is nothing more than an effort to vindicate the claims of Dr. W. J. G. Morton, to the discovery of Etherization, we have no other object in view than to point out and correct the following errors, viz. on page 26 it is stated that the American Society of Dental Surgeons was founded, "*above all to establish dental colleges throughout the United States,*" and that "the first institution of this kind established *under the auspices** of their society" . . . . . was the "Baltimore College of Dental Surgery." This is entirely wrong, the founders of the American Society had no such views. The Baltimore College was chartered by the Legislature of Maryland, during the session of January, 1839-40, six months before the society held its first meeting. (18th of August, 1840.) Indeed, the meeting of the society was not so much as *called*, until three months after the charter had been granted. And, thus it will be perceived, that the Baltimore College has the advantage of priority over all other institutions, (excepting the American Journal of Dental Science,) for the advancement of Dental Surgery.

On page 27, the following passage occurs: "During the next eighteen months, he diligently pursued the study of his profession, a portion of the time in Baltimore, the rest at the North. In 1842, *after his graduation*, he formed a co-partnership with Dr. Horace Wells . . . . "

We will not controvert the statement that "during the next eighteen months he diligently pursued the study of his profession," but we deny that the few weeks spent in Baltimore, were devoted to the systematic

*The Italics are our own.—Eds.

study of his profession. He was never so much as a matriculant of the College of Dental Surgery, far less did he graduate.

On page 28, the Society of Dental Surgeons is credited with having "established the American Journal of Dental Science." This is incorrect. The Journal was first published in 1839, and was nearly two years old when its management was conveyed over to an editorial committee, appointed by the Society.

On page 29, we find that "it was a habit, at the period of which we write, for *all* dentists, when fitting false teeth, to set them upon gold plate, placed directly upon the fangs of the old teeth, which were never removed." This is so far false, that among the hundred dentists of repute then practicing in the U. S., none were in the habit of applying false teeth in that manner. No manual of dentistry used at this time recommends any such practice.

It appears on page 45, "that, in treating a deformity, consisting of a hare-lip, cleft palate, deficient palatine arch and nasal septum," Dr. Morton employed "*his* new method of sustaining the plates for artificial teeth, namely, atmospheric pressure . . . . " His "new method of atmospheric pressure," as is well known to the profession, was practiced five or six years before, and was not originated by Dr. Morton. In conclusion, we have only to say, that in these remarks we do not wish it to be understood that we have impeached, by implication, Dr. Morton's claims to the honors of having first administered ether, to produce anæsthesia or to his skill as a practical dentist; our only object, as above stated, has been to correct glaring errors. For a more lengthy notice of the "trials," the reader is referred to an article, entitled "A Grain of Wheat in a Bushel of Chaff," in the February number of the "Knickerbocker Magazine."

*The Half-Yearly Abstract of the Medical Sciences.* Edited by W. H. Ranking, M. D. and C. B. Radcliffe, M. D. No. 28; July to December, 1858; 8vo.; 1859. Lindsay & Blakiston, Phila.

We have received the last number of this excellent work, and we take pleasure in recommending it to all students of the medical sciences. This No. contains a condensed account of a vast quantity of current professional matter; and we would particularly call the attention of dentists to the following: Article No. 25, on the shedding of teeth, by Salter; Article No. 43, on local narcotic injections in neuralgia; Article No. 93, on painless cauterization; the section consisting of four articles on anæsthetics, and to several special questions in surgery concerning the head and neck.

